# ML-Based Analysis of Particle Distributions in High-Intensity Laser Experiments: Role of Binning Strategy

**DOI:** 10.3390/e23010021

**Published:** 2020-12-25

**Authors:** Yury Rodimkov, Evgeny Efimenko, Valentin Volokitin, Elena Panova, Alexey Polovinkin, Iosif Meyerov, Arkady Gonoskov

**Affiliations:** 1 Department of Mathematical Software and Supercomputing Technologies, Lobachevsky University, 603950 Nizhni Novgorod, Russia; rodimkov@bk.ru (Y.R.); evgeny.efimenko@gmail.com (E.E.); valyav95@mail.ru (V.V.); alyona-gra98@yandex.ru (E.P.); 2 Institute of Applied Physics of the Russian Academy of Sciences, 603950 Nizhni Novgorod, Russia; 3 Mathematical Center, Lobachevsky University, 603950 Nizhni Novgorod, Russia; 4 Adv Learning Systems, TDAA, Intel, Chandler, AZ 85226, USA; alexey.polovinkin@gmail.com; 5 Department of Physics, University of Gothenburg, SE-41296 Gothenburg, Sweden

**Keywords:** laser physics, artificial neural networks, fully-connected neural networks

## Abstract

When entering the phase of big data processing and statistical inferences in experimental physics, the efficient use of machine learning methods may require optimal data preprocessing methods and, in particular, optimal balance between details and noise. In experimental studies of strong-field quantum electrodynamics with intense lasers, this balance concerns data binning for the observed distributions of particles and photons. Here we analyze the aspect of binning with respect to different machine learning methods (Support Vector Machine (SVM), Gradient Boosting Trees (GBT), Fully-Connected Neural Network (FCNN), Convolutional Neural Network (CNN)) using numerical simulations that mimic expected properties of upcoming experiments. We see that binning can crucially affect the performance of SVM and GBT, and, to a less extent, FCNN and CNN. This can be interpreted as the latter methods being able to effectively learn the optimal binning, discarding unnecessary information. Nevertheless, given limited training sets, the results indicate that the efficiency can be increased by optimizing the binning scale along with other hyperparameters. We present specific measurements of accuracy that can be useful for planning of experiments in the specified research area.

## 1. Introduction

In many experimental studies, the absence of clearly interpretable features in the measured data leads to the necessity of solving inverse problems for revealing the underlying properties of explored physical systems. Nevertheless, the problem may be intractable due to probabilistic or stochastic nature of the studied process or due to the presence of latent parameters that are not known within a necessary accuracy. These difficulties can be circumvented by the use of big data acquisition followed by statistical analysis carried out with the help of machine learning (ML) [1,2]. One way of arranging this is to develop a computational model that can reproduce the experimental data with sufficient accuracy for any given values of the unknown latent variables and the parameters that quantify the properties to be explored in an experiment. Using this computational model, we can generate possible outcomes of many experiments for various values of input parameters and train a machine learning algorithm to guess the values of these input parameters based on the simulated outcome. Even in the case of the probabilistic nature of the simulated process, the outcome may contain patterns that are sufficiently prominent to be learned and used by the ML model to unambiguously determine some of the latent parameters from the data measured in the experiment. Already this can be a crucial simplification for interpreting experimental results and for obtaining heuristic conclusions (see, for example, [3]). In less certain conditions and/or for more rigorous assessments, one can use the trained ML model as a generator that can dramatically increase the convergence rate of the approximate Bayesian computation (ABC) [4,5,6,7,8,9]. The application of the described routine can be useful in the experimental studies of strong-field quantum electrodynamics with the help of high-intensity lasers [10]. In many such experiments, beams of accelerated electrons collide with tightly focused laser pulses and the energy-angular distribution functions of the outgoing electrons and/or photons are measured [11,12]. Although some basic properties of certain processes can be studied via prominent features [13], the probabilistic nature of strong field quantum electrodynamics (SFQED) processes and uncontrollable (and unknown) variation of the interaction parameters (such as the impact parameter that quantifies the misalignment between the laser focus and the electron beam center) lead to the necessity of drawing statistical inferences from the data collected in a large series of experiments. ML methods can play an important role in the automatization of data processing for reinforcing not only experimental, but also theoretical studies [14].

In this paper, we assess the factor of binning, which is applied as a preprocessing of the measured distribution of particles. The choice of small bins leads to an increased level of noise, whereas the use of large bins reduces the noise at the cost of losing information due to reduced resolution. Although one can apply more advanced strategies, such as principle component analysis (PCA) and spectral filtering, the choice of optimal bin size can be sufficient in some cases, whereas various ML methods can differ in terms of their tolerance to this aspect. The consideration of a simple uniform binning strategy can be advantageous in sophisticated conditions, whereas the use of an optimal ML model can mitigate the effect of non-optimized binning. We analyze this aspect using a simplified computational model, which is designed to mimic the properties of particle distribution in the upcoming experiments with high-intensity lasers.

## 2. Problem Statement

The problem considered in the present paper is a simplified yet descriptive model of a numerical experiment that is closely related to novel experiments on radiation reaction [11,12]. In these experiments, head-on collisions of a high-intensity laser pulse with a high energy electron beam was used to find the experimental evidence on how the radiation reaction affects the electron dynamics. Here we analyze the employment of machine learning techniques to the problem of identification of latent parameters in such experiments. One known example is the impact parameter, which can vary uncontrollably from shot to shot if the alignment is not controlled sufficiently well [11]. In case of such a misalignment, the particles of the beam propagate aside of the pulse peak and effectively experience weaker electromagnetic fields. If we could identify the misalignment from the measured spectrum of electrons (or photons), we would be able to exclude unsuccessful shots and account for the misalignment in the remaining cases, thus making possible the further statistical analysis. To examine such a possibility, we model the effect of misalignment by the variation of the laser pulse amplitude in one-dimensional interaction process. Specifically, we aim to determine the laser pulse amplitude based on the spectra of an initially monoenergetic electron beam after interaction with this pulse in the presence of a quantum radiation reaction.

**Figure 1:**
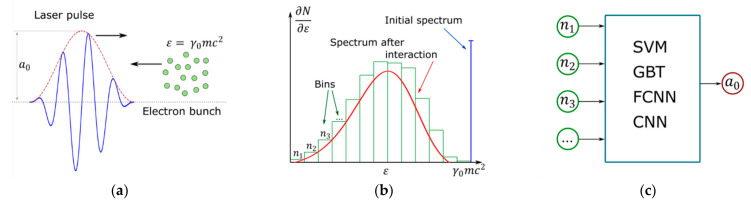
Schematic of numerical experiment. (**a**) Head-on collision of an ultra-intense laser pulse with an electron bunch. (**b**) Electron spectrum modification and binning to produce a resulting spectrum histogram. (**c**) A histogram serves as an input for different ML methods used to determine dimensionless amplitude of the laser pulse $a_{0}$.

The schematic description of the numerical experiment is as follows. An ultra-intense laser pulse propagates through a counter-propagating monoenergetic electron bunch, see Figure 1a. In the strong-field region, the effects of SFQED lead to a notable probability for an electron to emit one or several photons, and these events cause the corresponding loss of its kinetic energy. The process of photon emission is probabilistic, and in a single act of emission the electron may emit a photon, carrying away an arbitrary part of its kinetic energy. After the interaction, the energy distribution (spectrum) of electrons in the bunch has a finite width with a shift to lower energies with respect to the initial energy, see Figure 1b. To quantify electron spectra in a form suitable for a machine learning task, the full energy range from zero up to the initial energy is split into a number of bins, and the number of electrons in each bin is calculated. The resulting histogram representing the energetic spectrum of electrons is used as an input vector for the machine learning regression task, see Figure 1c. The problem of obtaining the pulse amplitude is solved by means of different machine learning techniques including fully connected and convolutional neural networks.

The interaction of the laser pulse with the electron bunch can be modeled by the following system of equations. The dynamics of electromagnetic field is governed by the Maxwell equations:

(1) $$\begin{array}{c} \frac{\partial \vec{E}}{\partial t}=-4\pi \vec{j}+c\nabla \times \vec{B}\\ \frac{\partial \vec{B}}{\partial t}=-c\nabla \times \vec{E} \end{array},$$

where $\vec{E},\;\vec{B}$ are the electric and magnetic fields, respectively, $\vec{j}$ is the current density, and c is the speed of light.

The motion of articles is described by Newton’s law of motion:

(2) $$\begin{array}{c} \frac{\partial \vec{p}}{\partial t}={\vec{F}}_{L}+{\vec{F}}_{RR};\;{\vec{F}}_{L}=e\cdot \left(\vec{E}+\frac{1}{c}\vec{v}\times \vec{B}\right)\\ \frac{\partial \vec{r}}{\partial t}=\vec{v}=\frac{\vec{p}}{m}{\left(1+\frac{p^{2}}{m^{2}c^{2}}\right)}^{-1/2}, \end{array}$$

where $\vec{r},\;\vec{p},\;\vec{v}$ are the position, momentum, and velocity of the particle, m, e are its mass and charge, respectively, ${\vec{F}}_{L}$ is the Lorentz force due to electromagnetic field acting on the particle. The term ${\vec{F}}_{RR}$ provides the semiclassical description of the radiation reaction by instantaneous changes of momentum (the recoil due to photon emission) that occur probabilistically with the rate computed within SFQED (see, for example, [15]).

The scheme of the numerical experiment is close to the one used in Ref. [16]. Initially, the electrons in the bunch have the energy $\varepsilon_{0}=mc^{2}\gamma_{0}$, where $\gamma_{0}=1000$ is the relativistic Lorentz-factor. The number of electrons in the bunch is varied in the experiments from 25 to 100,000. As the number of electrons in the bunch is sufficiently small, we neglect all types of their influence on the laser beam, such as the refraction and the depletion of the electromagnetic fields of the pulse. We also neglect the direct electron–electron interactions inside the bunch. With these simplifications we can consider the interaction between each electron and the laser pulse independently.

For simplicity, the one-dimensional problem is studied and the laser field is set as a short laser pulse propagating in the x direction:

(3) $$E_{y}=-B_{z}=a_{0}E_{\mathrm{rel}}\sin^{2}\left(\pi \frac{x+\mathrm{ct}}{\lambda \;}\right)\sin\left(2\pi \frac{x+\mathrm{ct}}{\lambda \;}\right),$$

where λ=1 μm is the laser wavelength, and $a_{0}$ is the dimensionless amplitude in relativistic units $E_{\mathrm{rel}}=\frac{2{\pi \mathrm{mc}}^{2}}{\lambda e}$. The pulse is evolved according to Equation (3) over the total simulation time T=L/c with the number of time steps equal to 100. The dimensionless amplitude $a_{0}\;$is varied from 10 to 1000. This covers a wide range of intensities from 10^20^ W/cm^2^, where radiation losses are weak and radiative friction can be treated classically, up to 10^24^ W/cm^2^, where radiative friction becomes essentially probabilistic. In the latter case, the electrons can lose a major part of their energy, and a significant spectrum broadening is observed.

The described problem is modeled using the Hi-Chi open-source framework [17]. The photon emission and electron recoil are accounted for in the following way. On each time step for each electron, we generate a uniformly distributed value $\;\delta =\frac{\hbar \omega }{mc^{2}\gamma }$, which is the ratio of photon energy to the full energy of the original particle $\varepsilon =mc^{2}\gamma$, and then we sample the new photon with probability density $P\left(\delta \right)$:

(4) $$P\left(\delta \right)=\left[\Delta t\frac{e^{2}mc}{\hbar^{2}}\right]\cdot \frac{\sqrt{3}}{2\pi }\cdot \frac{\chi }{\gamma }\cdot \frac{1-\delta }{\delta }\cdot \left(F\left(z_{q}\right)+\frac{3}{2}\delta \chi z_{q}G\left(z_{q}\right)\right),$$

where ℏ is the reduced Planck constant, Δt is the time step, $F\left(x\right)$ and $G\left(x\right)$ are the first and second synchrotron functions, $\;z_{q}=\frac{2}{3}\chi^{-1}\frac{\delta }{1-\delta }$, and $\chi \equiv \frac{e\hbar }{m^{3}c^{4}}\sqrt{{\left(\frac{\varepsilon \vec{E}}{c}+\vec{p}\times \vec{B}\right)}^{2}-{\left(\vec{p}\cdot \vec{E}\right)}^{2}}$ is a dimensionless parameter characterizing the transverse acceleration of the particle in the field. For electrons, this parameter can be calculated as $\chi =\gamma \frac{H_{eff}}{E_{s}}$, where $E_{s}=\frac{m^{2}c^{3}}{e\hbar }$ is the Schwinger field and $H_{eff}$ is the effective field that acts on the particle. The generated photon is assumed to have the same direction of propagation as the parent particle. An electron’s momentum and energy are updated accordingly. We consider laser field intensities achievable on existing laser facilities. We neglect the effect of Breit–Wheeler pair production. We also assume that the electron bunch duration is sufficiently short so that we can neglect the interactions of emitted photons with the electron bunch after they have been emitted. After all electrons have interacted with the laser pulse, we retrieve the electron energy distribution for the given amplitude $a_{0}$. Since the process of photon emission is probabilistic and for a small number of electrons the energy distribution can be noisy, we collected several realizations for each $a_{0}$.

In the next stage, we divided the whole energy range from 0 to $mc^{2}\gamma_{0}$ into a number of bins. For each realization, we counted the number of electrons in each bin, denoted as $n_{i}$ for the i-th bin in Figure 1b,c. We generated a dataset consisting of a vector of$\;n_{i}$ as a feature vector and $a_{0}$ as a label, and trained ML models using Support Vector Machine (SVM), Gradient Boosting Trees (GBT), Fully-Connected Neural Network (FCNN), and Convolutional Neural Network (CNN) on generated data to solve the regression problem of estimating $a_{0}$ based on the histogram of electrons’ energy spectra. The aim of this paper is to examine the role of the binning strategy, so the accuracy of numerical methods was investigated with respect to the combination of the number of bins and the number of electrons per bin. After dimensionality reduction by means of the principal component analysis method and fine-tuning, we found the most relevant model and analyzed its results.

## 3. Methods

### 3.1. Hi-Chi Project Overview

The project High-Intensity Collisions and Interactions (Hi-Chi) is an open-source collection of Python-controlled tools for performing simulations and data analysis in the research area of strong-field particle and plasma physics. The project is intended to offer an environment for testing, benchmarking, and aggregative use of individual components, ranging from basic routines to supercomputer codes. The components are being developed in C++ and optimized for state-of-the-art high-performance CPUs. In this way, the project combines the flexibility of Python and the efficiency of resource-intensive computations at the C++ level, achieving high performance using either desktops or supercomputers.

**Figure 2:**
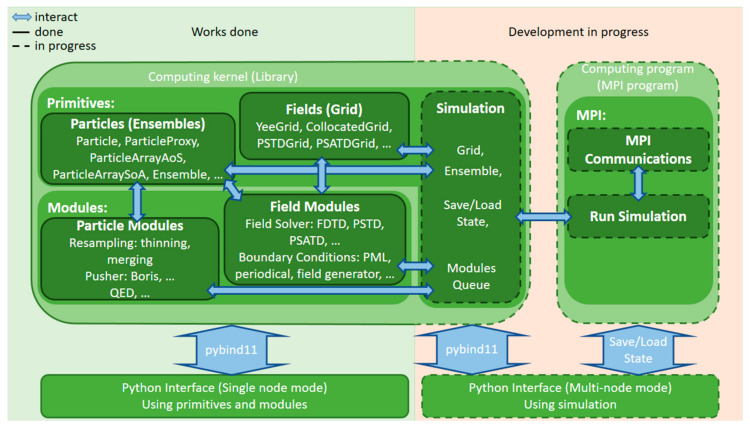
The interaction scheme of the High-Intensity Collisions and Interactions (Hi-Chi) modules.

A high-level architecture of the project is depicted in Figure 2. The project’s architecture is designed as an independent set of primitives and modules that can interact with each other. Currently, there are two types of modules: (I) Working with an electromagnetic field and (II) interacting with ensembles of particles. Modules of the first type include finite-difference time-domain (FDTD) [18] and spectral (PSTD, PSATD) field solvers [19,20,21,22], several implementations of boundary conditions (periodic, PML, field generator), transformations of electromagnetic field (rotation, shift, scaling, etc.). Modules of the second type include several particle motion equations solvers (e.g., the Boris method), a number of particle resampling methods (various particles thinning and merging techniques [23]), and a module taking into account quantum electrodynamic effects (the QED module) [15]. Each module interacts with relevant primitives. Thus, the field solvers are associated with collocated and staggered grids capable of performing field interpolation at any point of a computational domain. For this purpose, the CIC and TSC form factors are currently supported. The particle pushers work with ensembles of particles which are stored employing the Structure of Arrays (SoA) or Array of Structures (AoS) patterns. All C++ classes and objects are exported from C++ to Python by means of the pybind11 software [17].

The Hi-Chi implementation is based on the experience of the development of the high-performance plasma simulation PICADOR code [24,25] and currently employs shared-memory parallelism using the OpenMP technology. Main computational kernels are optimized for state-of-the-art CPUs including vectorization and parallelization of performance-critical computational loops, cache optimizations, and NUMA (Non-Uniform Memory Access) [26] optimizations. The code is under ongoing modifications and improvement. One of the main directions of further development is the creation of a distributed version of the code that allows you to utilize a supercomputer through the use of MPI technology. Note that the interaction of Python and C++ in a distributed mode is not straightforward. However, Python and C++ modules can interact, saving and loading states in the file system. For this purpose, the user-defined configuration can be saved in the file system and a chosen number of MPI processes will be launched. Then, each process can download the configuration from the file system, perform calculations, and save the final results. The results can be further read and processed by a Python-based control program. The distributed version of the code is under development. The code is publicly available (see Supplementary Materials section for the details).

### 3.2. Data Generation

We collected data as follows. Firstly, we performed numerical simulations with the peak amplitude $a_{0}$ in the range $\left[10;\;1000\right]$ and with N=100,000 electrons up to time T=L/c, integrating the electron motion equations and taking into account the QED effects. The resulting data array, hereinafter referred to as DATA, contained N energies for each $a_{0}$. It was used to randomly sample the resulting values with their subsequent aggregation into $N_{b}$ bins. After sampling, all values were normalized to the range [0; 1] to improve the performance of training machine learning models.

Secondly, we used the DATA array to train several machine learning models and test how their accuracies depend on the number of electrons involved in the numerical simulations. In this regard, we fixed different values of the number of bins $N_{b}$ and the average number of electrons per bin $N_{e}$ and randomly selected $N_{b}\times N_{e}$ electrons from the DATA array. The values $N_{b}$ and $N_{e}$ varied in the range $\left[5;\;2000\right]$, while the total number of electrons varied in the range $\left[25;\;20,000\right]$. All samples were taken without repetitions. When forming the training dataset, the specified procedure was performed three times, while at the stage of creating the validation and test samples it was done only once.

### 3.3. Machine Learning Techniques

We evaluated and compared several state-of-the-art supervised machine learning algorithms to solve the regression problem for the estimation of $a_{0}$ based on the histogram of electron spectra.

Support vector regression machine [27] (evolution of support vector machine (SVM) [28] for classification problems) is a powerful algorithm that can balance tolerance to the errors, both through setting an acceptable error margin and through tuning the cost of falling outside this acceptable error margin. One of the main SVM advantages is the use of kernels for learning linear predictors in high dimensional feature spaces that allows us to handle high-dimensional problems effectively.

Gradient boosting trees (GBT) [29] is an ensemble of decision trees [30] where every new tree is built using the data from previously learnt trees. At each iteration of GBT, a new tree is fitted to the generalized residuals with respect to a loss function. The GBT algorithm can deal with both classification and a regression problem, works with mixed type data, effectively processes missing data, and is invariant to monotonic transformations of the input variables. All these factors make GBT one of the most accurate and universal supervised machine learning algorithms.

Neural networks and their applications have been widely developed recently due to explosive growth of computational capabilities and accumulation of a large amount of data necessary for effective training of these models. According to Cybenko theorem [31], a feed-forward neural network with one hidden layer can approximate any continuous function of many variables with any given precision. In recent studies, in particular [32], it has been proven that any Lebesgue integrable function of many variables can be approximated by a fully connected neural network with ReLU activations. In this work, we also consider convolutional neural networks [33] that consider local special data dependencies.

## 4. Experimental Results

### 4.1. Methodology

The experimental part of the paper is as follows. Firstly, we run some preliminary experiments to determine the appropriate hyperparameter values for each of the machine learning methods used (SVM, GBT, CNN, FCNN). Having temporarily fixed these parameters, we empirically investigate how the accuracy of solving the problem depends on the number of bins and electrons involved in the numerical simulation. We consider from 5 to 2000 bins and from 5 to 2000 electrons per bin. For each point in the $\left\{N_{b};N_{e}\right\}$ parameter space, we train ML models. Stopping the training of neural networks is carried out based on the error in the validation dataset, and the accuracy is estimated using the test dataset. Realizing that the chosen “generic” hyperparameter values may not be optimal, we selectively examine some configurations $\left\{N_{b};\;N_{e}\right\}$ by manually adjusting the hyperparameters. Indeed, experiments show that accuracy can be improved by fine-tuning, but we did not find any dramatic changes.

The main idea behind the series of experiments described above is to gain an intuition as to how accurately specific machine learning methods can solve a given problem, to understand which of them are most promising for further tuning, and also to establish how stable the results are when the number of electrons and bins decreases. Based on these experiments, we choose the most promising configurations $\left\{N_{b};\;N_{e}\right\}$ and investigate them in more detail, adjusting the parameters to improve the results.

Finally, we examine the feasibility of feature selection and dimensionality reduction techniques. The feature selection does not lead to an improvement in the results, while the dimensionality reduction employing the principal component method makes it possible to reduce the number of features and simplify the architecture of the artificial neural networks, with relevant accuracy.

### 4.2. Results and Discussion

#### 4.2.1. How Accuracy of ML Models Depends on the Number of Bins and the Number of Electrons per Bin?

Firstly, we performed massive experiments to establish how the accuracy of reconstruction of the peak amplitude of a laser pulse depends on the parameters $\left\{N_{b};\;N_{e}\right\}$. Given that a full consideration of all relevant combinations of hyperparameters for four machine learning methods for each pair $\left\{N_{b};\;N_{e}\right\}$ would require huge computational resources, we performed preliminary experiments for some pairs, and then fixed the parameters as follows. We employed the XGBRegressor method from the XGBoost library [34] and the SVR method from the scikit-learn library [35] as the implementation of the GBT and SVM methods, respectively. In the GBT method, we used 110 trees of depth 5, the learning rate was set to 6 × 10^−2^ [36]. In the SVM method, we used the radial basis function (RBF) kernel, the epsilon was equal to 1 × 10^−3^ [37]. The default values were used for the rest of the parameters.

The parameters of neural networks in the CNN and FCNN methods were selected by optimizing the error on the validation set taking into account the dimension of the input vector. We employed the following architectures and considered them in the specified ranges of hyperparameters (the selected optimal parameters are detailed in Section 4.2.2): FCNN with 3–5 hidden layers, CNN with 1–6 convolution layers with a kernel of size 3 at the beginning, and 2–4 fully connected hidden layers at the end. The numbers of neurons in the fully-connected layers were taken from the range 4–200. We used the Adam optimizer from the Keras framework [38] with default parameters and the ReLU activation function. The numbers of neurons in each layer were selected based on the dimension of the input data. For different pairs $\left\{N_{b};\;N_{e}\right\}$ the architectures and parameters of the neural networks could be slightly different in order to improve the accuracy. Further, for the most promising combinations $\left\{N_{b};\;N_{e}\right\}$ we fine-tuned the hyperparameters for all the methods used. The best found configurations are given in Section 4.2.2.

**Figure 3:**
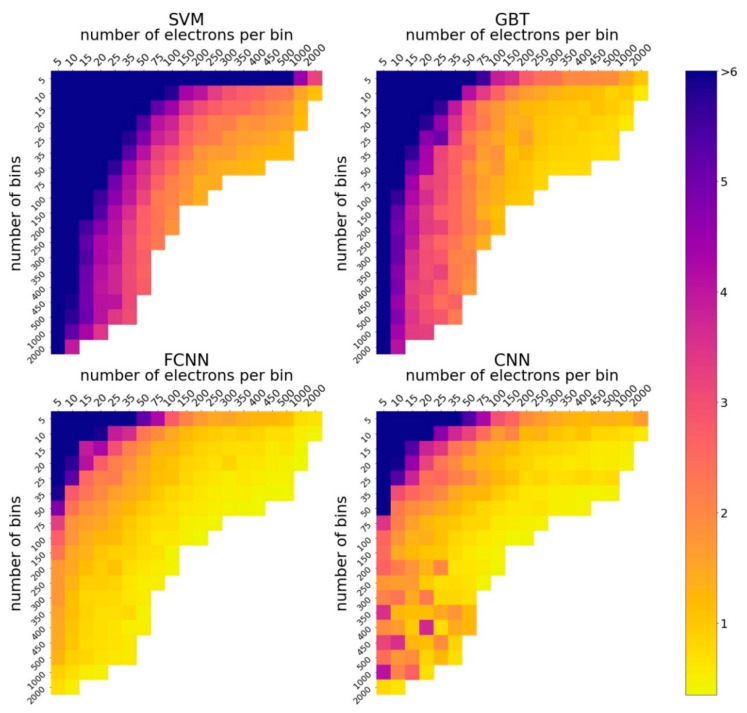
Heat maps demonstrate how the accuracy of the Support Vector Machine (SVM), Gradient Boosting Trees (GBT), Fully-Connected Neural Network (FCNN), Convolutional Neural Network (CNN) methods in reconstructing the peak amplitude of a laser pulse depends on the number of bins and the number of electrons per bin. Accuracy is given as a percentage of the mean relative error. Blue squares correspond to a large error, yellow squares to a small error.

Figure 3 shows how four ML methods reconstruct the peak amplitude of a laser pulse depending on the number of bins $N_{b}$ and the number of electrons $N_{e}$, while $N_{b}\in \left[5;\;2000\right]$, $N_{b}\in \left[5;\;2000\right]$, and the total number of electrons $N_{b}\times N_{e}$ varies in the range [25; 20,000]. The results show, as expected, that an increase in the number of electrons usually leads to a decrease in the error. We can also compare the methods and conclude how the parameters $\left\{N_{b};\;N_{e}\right\}$ should be chosen.

The FCNN demonstrates perfect stability in terms of accuracy when a reasonable configuration is chosen, even with the fixed network architecture and parameters. The CNN shows good accuracy, but the results seem less stable. We observe that for a small number of electrons and a large number of bins, the accuracy varies over a wide range, even with a small change in the parameters. The SVM and GBT methods are inferior in accuracy to neural networks, but still show reasonable results.

**Figure 4:**
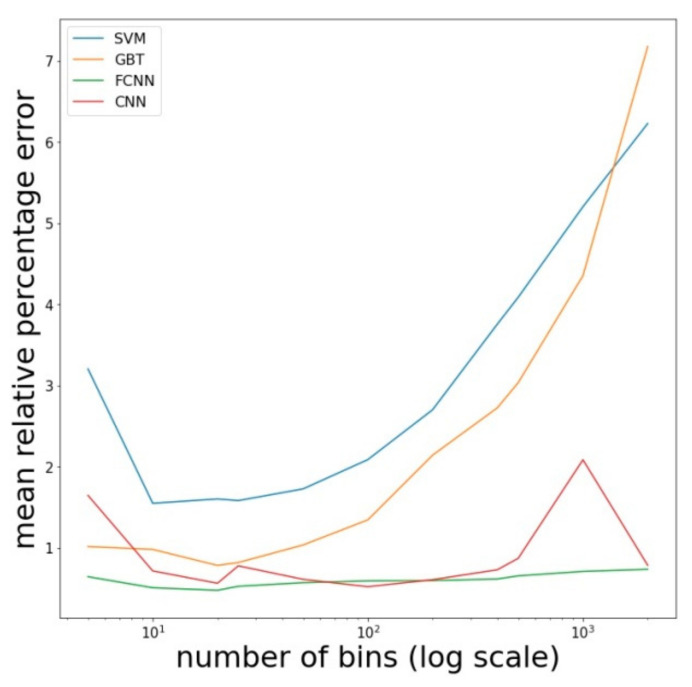
The dependence of the mean relative percentage error for the four considered machine learning methods (SVM, GBT, FCNN, CNN) on the number of bins used to build the histogram. The number of electrons in the experiment is equal to 10,000.

Next, we fix the relevant number of electrons in a numerical experiment, equal to 10,000, and analyze how the error changes when the number of bins increases (Figure 4). It turned out that for the GBT method and FCNN, the optimal number of bins is equal to 20. For the SVM method, it is equal to 10, but the accuracy for 10 bins only slightly exceeds the accuracy for 20 bins. Thus, the considered methods work best with approximately the same small number of bins. For the CNN, 100 bins are optimal. Based on these results, we fine-tuned the models. The results obtained in this case, as well as the optimal parameter configurations, are described below.

#### 4.2.2. Optimal Configuration of the Parameters

This section describes the hyperparameters of the models in their best configurations. Firstly, empirically optimal parameters for GBT and SVM methods are considered. We tune the parameters of cross-validated XGBRegressor method from the XGBoost library [34] and the SVR method from the scikit-learn library [35] as the implementation of the GBT and SVM methods, respectively. We found that the GBT method performed best when using 110 trees with maximum tree depth equal to 6 and a learning rate of 0.1, without regularization [36]. The SVM method showed the best results when using the radial basis function (RBF) kernel, the L2 regularization with parameter 30, and the epsilon equal to 3 × 10^−4^ [37]. The default values were used for other parameters.

Secondly, we customize the architecture and parameters of artificial neural networks. We employ a fully-connected model with 5 hidden layers. The first hidden layer contains 100 neurons with the ReLU activation function, followed by a layer with 75 neurons and the sigmoid activation function. The last three hidden layers use the ReLU activation function and contain 64, 16, and 4 neurons, respectively. The model was trained for 1420 epochs, with the Adam optimizer [39] with the learning rate of 1 × 10^−3^. By analogy with FCNN, various options for combining layers with different numbers of neurons were considered for CNN. We employ two convolutional layers containing 1 and 3 convolutions, respectively, followed by a pooling layer with the size of 2. Further, the same combination of layers was used with the difference that the number of kernels was set equal to 3 and 9, respectively. For all convolutional layers, the convolution size is 3, with the ReLU activation function. Further, 4 fully connected layers are used, containing 96, 64, 16, and 4 neurons with the following activation functions: Sigmoid, sigmoid, ReLU, and ReLU, respectively. The model was trained for 1520 epochs. We used the Adam optimizer with the learning rate of 3 × 10^−4^.

Then, we employ the PCA method from the scikit-learn library [40]. We found that the first 5 principal components explain 98 percent of the variance in the original data. After that, we customize a fully connected neural network with 5 hidden layers. The first 3 layers contain 10 neurons, followed by 2 layers with 8 and 4 neurons, respectively. The ReLU activation functions are used. The neural network was trained for 2800 epochs. We used the Adam optimizer with a learning rate of 6 × 10^−4^. All models were trained in batches of 32 objects. For training, the mean absolute error was used. The Adam optimizer used the default parameters from the Keras framework [38], except for the learning rate parameter, the values of which are given above.

#### 4.2.3. Final Comparison

The results of a comparative analysis of models created by machine learning methods for the optimal configurations of parameters are shown in Table 1. It turned out that in most cases, fine-tuning of the hyperparameters of the methods led to some increase in accuracy. At the same time, the achieved gain is not dramatic, which indicates that it is enough to choose the reasonable values of the parameters. Experiments have shown that artificial neural networks solve the problem of reconstructing the peak amplitude of a laser pulse with sufficiently higher accuracy. The SVM method loses out to deep learning methods by about a factor of two in terms of the average absolute and average relative error. The GBT method shows accuracy close to that of neural networks. However, unlike artificial neural networks, the GBT and SVM methods, with a small number of objects, can yield an error of 5–10%, which can be critical for practical use. The PCA method allowed us to reduce the size of the network and decrease the run time while maintaining a reasonable accuracy of the amplitude reconstruction. We applied this method to data for a fully connected neural network and selected 5 principal components, on which another fully connected neural network was trained. New features are not correlated, which also improves the neural network training procedure. The new data explains 98 percent of the variance in the original data.

**Figure 5:**
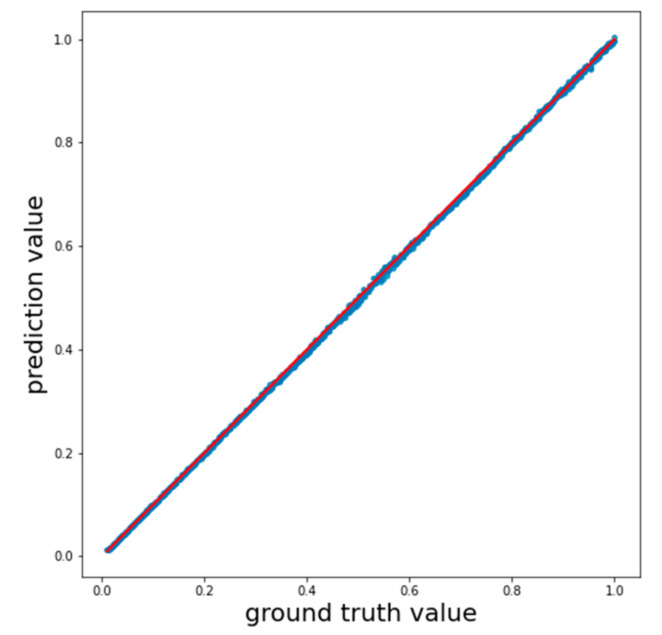
Correlation of the exact and predicted values when using the FCNN model. Points correspond to pairs of exact and predicted values. The red line is the linear function y=x.

Figure 5 shows the correlation between exact and predicted values for a fully connected neural network. The points are almost perfectly fitted by the linear function y=x, shown in red, which corresponds to the close to 1 value of the coefficient of determination. The rest of the methods show similar results (Table 1).

**Figure 6:**
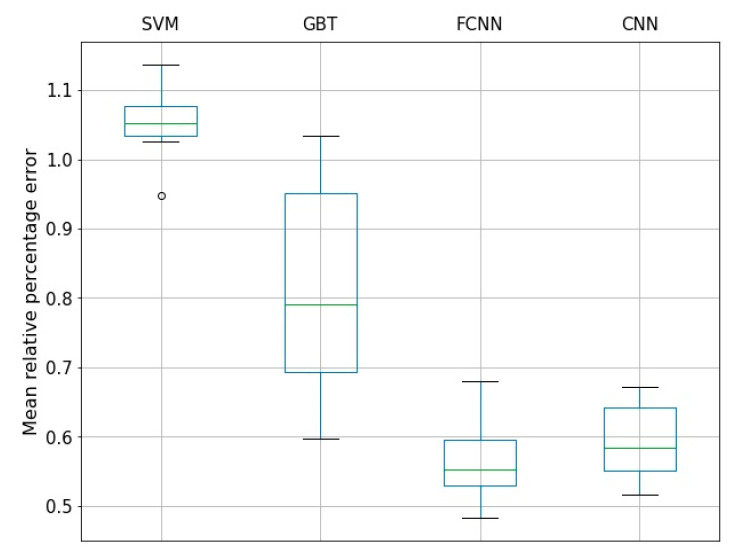
Distribution of mean relative percentage errors for 4 ML methods: SVM, GBT, FCNN, CNN.

Lastly, we run a *t*-test to compare ML models in their optimal configurations in terms of accuracy. To do this, we combined training and test samples for the fine-tuned ML models. Next, we randomly divided the obtained data into new training and test samples 10 times and calculated the accuracy of the models. The results are presented below (Figure 6). The ML models were further sorted by accuracy and compared by the paired *t*-test (the most accurate model was compared with the second one, the second model with the third one, and so on). As a null hypothesis, it was assumed that the methods are indistinguishable in accuracy. The *p*-value was equal to 0.05. The *t*-test results showed that FCNN, CNN methods work better on this problem than GBT, and SVM shows the worst result.

## 5. Conclusions

In this work, we considered the effect of binning strategy on the accuracy of several ML models applied to a test problem that models the needs of the upcoming experiments on the SFQED effects. We varied the size of bins used for the construction of the input vector from the energy spectra that can presumably be measured with high resolution. The limit of small bins (i.e., large input vectors) corresponds to a high level of noise, whereas the use of large bins (i.e., small input vectors) implies the loss of information. The results indicate that SVM and GBT are more sensitive to the choice of the bin size than FCNN and CNN, but all the considered ML models can be configured to achieve a reasonably good accuracy in our tests. The studies carried out do not guarantee the success of solving more complex problems. However, they show the prospects for continuing work in this direction. In the future, we plan to consider problems closer to state-of-the-art physical experiments based on the experience gained.

One of the potential directions for further development is the use of new approaches to dimensionality reduction, in particular, non-linear PCA options based on principal manifolds [41]. We also plan to pay special attention to the issues of reliability and explainability of the results obtained using artificial neural networks. We believe that these questions are extremely important for planning future experiments. In the model problem considered in this paper, we see that FCNN shows good accuracy with an appropriate binning strategy in a wide range of parameters. However, the question of whether this effect will persist in more complex problems remains open.

## Figures and Tables

**Table 1:** Accuracy of the fine-tuned machine learning methods for solving the peak amplitude reconstruction problem with 10,000 electrons for one feature vector.

Measure	SVM	GBT	FCNN	CNN	PCA+FCNN
Mean absolute error	4.050	2.453	1.784	1.827	2.000
Mean relative percentage error	1.062	0.661	0.512	0.496	0.709
Coefficient of determination	0.99930	0.99967	0.99993	0.99992	0.99991

## Data Availability

The data generated and analyzed in this study are publicly available in https://github.com/hi-chi/Machine-Learning (the relevant examples are located in the “Amplitude Reconstruction” folder).

## Supplementary Materials

The Hi-Chi project is available online at https://github.com/hi-chi/pyHiChi. The data and scripts required to reproduce the numerical results may be downloaded from https://github.com/hi-chi/Machine-Learning (the relevant examples are located in the “Amplitude Reconstruction” folder).
